# Future of sentinel node biopsy in ovarian cancer

**DOI:** 10.1097/CCO.0000000000001058

**Published:** 2024-07-16

**Authors:** Nicolò Bizzarri, Camilla Nero, Stefano Di Berardino, Giovanni Scambia, Anna Fagotti

**Affiliations:** a UOC Ginecologia Oncologica, Dipartimento di Scienze della Salute della Donna, del Bambino e di Sanità Pubblica, Fondazione Policlinico Universitario A. Gemelli, IRCCS; b Università Cattolica del Sacro Cuore, Rome, Italy

**Keywords:** accuracy, detection, lymphadenectomy, ovarian cancer, sentinel lymph node, surgical staging

## Abstract

**Purpose of review:**

The rationale on the use of sentinel lymph node (SLN) biopsy in the surgical staging of apparent early-stage ovarian cancer (OC) is supported by the fact that diagnostic and prognostic role of systematic staging lymphadenectomy has been determined but its therapeutic significance is still matter of controversy. Moreover, SLN biopsy represents an option to decrease intra- and postoperative morbidity. The present review aims to provide an overview on the current and future role of SLN in OC.

**Recent findings:**

Most recent evidence shows that the overall mean per patient SLN detection rate in case of indocyanine green (ICG) alone was 58.6% compared with 95% in case of ICG + technetium, and with 52.9% in case of technetium alone or in combination with blue dye (*P* < 0.001). Site of injection has been reported to be in both ovarian ligaments in majority of studies (utero-ovarian ligament and infundibulo-pelvic ligament), before or after ovarian mass removal, at time of primary or re-staging surgery and by minimally invasive or open approach. Cervical injection has been recently proposed to replace utero-ovarian injection. SLN detection rate in patients with confirmed ovarian malignancy varied across different studies ranging between 9.1% and 91.3% for the injection in the utero-ovarian ligament and migration to pelvic lymph nodes and between 27.3% and 100% for the injection in the infundibulo-pelvic ligament and migration to para-aortic lymph nodes. No intra- or postoperative complication could be attributed directly to SLN biopsy. The sensitivity and the accuracy of SLN in detecting lymphatic metastasis ranged between 73.3–100% and 96–100%, respectively. In up to 40% of positive SLNs, largest metastatic deposit was classified as micro-metastasis or isolated tumor cells, which would have been missed without ultrastaging protocol.

**Summary:**

SLN biopsy represents a promising tool to assess lymph node status in apparent early-stage OC. The type and volume of injected tracer need to be considered as appear to affect SLN detection rate. Ultrastaging protocol is essential to detect low volume metastasis. Sensitivity and accuracy of SLN biopsy are encouraging, providing tracer injection in both uterine and ovarian ligaments.

## INTRODUCTION

Ovarian cancer (OC) is the most lethal gynecological cancer with approximately 314 000 diagnoses and 207 000 deaths worldwide in 2020 [1]. In 15–20% of cases it is diagnosed at an apparent early-stage of disease and comprehensive surgical staging including systematic pelvic and para-aortic lymphadenectomy is required in epithelial histologies [2]. The concept of sentinel lymph node (SLN) has been successfully implemented in uterine and vulvar cancers with international guidelines endorsing its use as method for assessing lymph node status [3–5]. Compared to systematic lymphadenectomy, SLN offers the advantage of reducing intra- and post-operative morbidity and to increase the diagnostic accuracy thanks to the ultrastaging method, which allows the detection of low-volume metastases [6]. SLN concept in OC staging has been investigated later in time compared with vulvar and uterine cancers [7]. Its use in the surgical staging of apparent early-stage OC is supported by the fact that therapeutic role of lymphadenectomy is still matter of controversy [8^▪^]. Nevertheless, the staging and prognostic value of nodal status remains pivotal in indicating adjuvant therapy and maintenance treatment.

The present review aims to provide an overview on the current role of SLN in OC, focusing on the studies published in English language in the last five years. Furthermore, future perspectives regarding its application have also been delineated. 

**Box 1:**
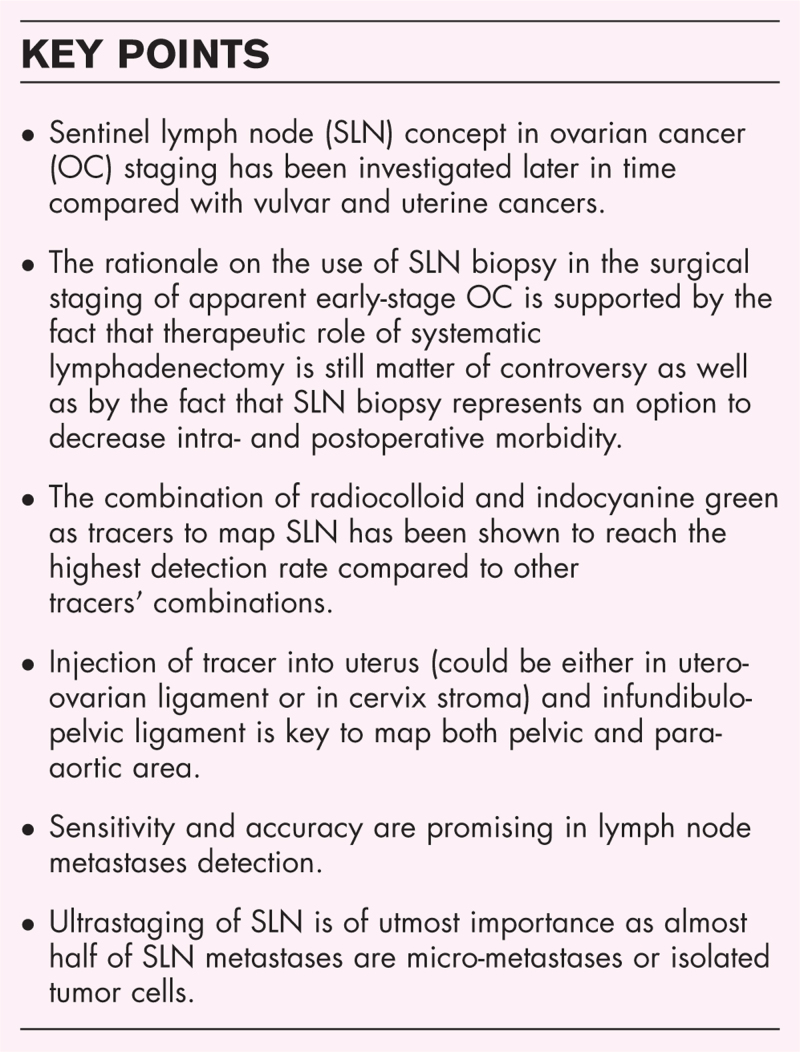
no caption available

## TYPE OF TRACER

First descriptions of SLN technique in OC used 99mTc radioisotope and blue dye alone or in combination [9^▪▪^,^10^]. More recently, indocyanine green (ICG) has been proposed to map SLN in OC after its large application in SLN detection in uterine cancers with demonstrated superiority when compared to other tracers [11,12]. Nevertheless, the use of ICG alone [13^▪▪^,^14^^▪▪^] has been associated with lower SLN detection rates compared with ICG + radioisotope [15^▪^,16^▪^] but similar to those obtained with radioisotope ± blue dye [17^▪^,18^▪^]. In particular, the overall mean (per patient) SLN detection rate in case of ICG alone was reported to be 58.6% (99/169) [13^▪▪^] compared with 95% (57/60) in case of ICG + technetium [14^▪▪^,15^▪^], and with 52.9% (9/17) in case of technetium alone or in combination with blue dye [16^▪^,17^▪^] (*P* < 0.001). Despite this difference, a recently published meta-analysis did not find a statistically significant difference in SLN detection rate when different tracers were compared, but a large heterogeneity between the included studies was reported [18^▪^]. Volume of tracer injection was also different across the studies, ranging from 0.2 ml to 2 ml per site of injection [13^▪▪^,^14^^▪▪^,15^▪^,16^▪^,17^▪^]. Characteristics of the most recent studies on SLN use in OC are reported in Table 1.

**Table 1:** Studies on SLN biopsy in apparent early-stage ovarian cancer (published in the last 5 years)

Authors	Year	Number of patients^a^	Surgical approach	Setting	Timing of injection	Tracer	Site of injection	SLN detection	SLN ultrastaging	Positive SLN	Sensitivity	Accuracy	Positive LN^b^
Lago *et al.*[14^▪▪^]	2021	30	12 MIS (40%) 18 LPT (60%)	18 PS (60%) 12 RS (40%)	AA	0.2 ml of Tc-99m albumin colloid (37 MBq) + 0.5 ml of ICG (1.25 mg/ml)	23 UO ligaments (7 previously performed hysterectomy) 30 IP ligaments	30/30 (100%) Overall^c^ 21/23 (91.3%) Pelvic region 27/30 (90%) Para-aortic region	Yes	1/30 (3.3%) Overall 0/21 (0%) Pelvic region 1/27 (3.7%) Para-aortic region	100%	100%	2/30 (6.7%) Overall 0/23 (0%) Pelvic region 2/30 (6.7%) Para-aortic region
Laven *et al.*[16^▪^]	2021	11	11 LPT (100%)	8 PS (72.7%) 3 RS (27.3%)	AA	0.15 ml of Tc- 99m albumin nanocolloid (20 MBq) + 0.2 ml blue dye	11 UO ligaments (no previous hysterectomy) 11 IP ligaments	3/11 (27.3%) Overall^c^ 1/11 (9.1%) Pelvic region 3/11 (27.3%) Para-aortic region	Yes	0/3 (0%) Overall 0/1 (0%) Pelvic region 0/3 (0%) Para-aortic region	100%	100%	1/11 (9.1%) Overall NA Pelvic region NA Para-aortic region
Nero *et al.* (SELLY)^d^[13^▪▪^]	2023	169	95 MIS (56.2%) 74 LPT (43.8%)	120 PS (71.0%) 49 RS (29.0%)	AA	2 ml of IGC (1.25 mg/ml)	51 UO ligaments (8 previously performed hysterectomy) 169 IP ligaments	99/169 (58.6%) Overall^c^ 26/51 (51.0%) Pelvic region 93/169 (55.0%) Para-aortic region	Yes	11/99 (11.1%) Overall 1/26 (3.8%) Pelvic region 10/93 (10.7%) Para-aortic region	73.3%	96%	20/169 (11.8%) Overall 4/163 (2.4%) Pelvic region 18/169 (10.6%) Para-aortic region
Agusti *et al.* (MELISA) [15^▪^]	2023	30	7 MIS (23.3%) 23 LPT (76.7%)	18 PS (60%) 12 RS (40%)	BA and AA	0.2 ml of Tc-99m albumin colloid (37 MBq) + 0.2 ml of ICG (1.25 mg/ml)	27 UO (1 previously performed hysterectomy) (In other 2 cases UO ligament not injected for adnexectomy en bloc with hysterectomy) 30 IP ligaments	27/30 (90%) Overall^c^ 13/29 (44.8%) Pelvic region 26/30 (86.7%) Para-aortic region	Yes	5/30 (16.7%) Overall 2/13 (15.4%) Pelvic region 3/26 (11.5%) Para-aortic region	100%	100%	5/30 (9.1%) Overall 3/29 (10.3%) Pelvic region 3/30 (10%) Para-aortic region
Ataei Nakhaei *et al.*[17^▪^]	2024	6	6 LPT (100%)	6 PS (100%)	BA	0.2–0.5 l of Tc-99m-Phytate (18.5 Bq)	6 UO ligaments (no previous hysterectomy) 6 IP ligaments	6/6 (100%) Overall^c^ 4/6 (66.7%) Pelvic region 6/6 (100%) Para-aortic region	No	0/6 (0%) Overall 0/4 (0%) Pelvic region 0/6 (0%) Para-aortic region	100%	100%	0/6 (0%) Overall 0/6 (0%) Pelvic region 0/6 (0%) Para-aortic region

AA, after adnexectomy; BA, before adnexectomy; IP, infundibulo-pelvic ligament; LN, lymph node; LPT, laparotomy; MIS, minimally invasive surgery; NA, not available from original article; PS, primary surgery; RS, restaging surgery; SLN, sentinel lymph node; UO, utero-ovarian ligament.

a Ovarian cancer only.

b Including both SLN and non-SLN (one patient could have had both positive pelvic and para-aortic node).

c At least one SLN between pelvic and para-aortic region.

d Data on surgical approach, setting and detection per site of injection were not reported in the original article.

## SITE AND TIMING OF INJECTION

Anatomical studies on the lymphatic flow of the ovary demonstrated infundibulo-pelvic ligament (suspensory ligament of the ovary), utero-ovarian ligament (ligamentum ovarii proprium) and (in a lower extent) round ligament as the three main drainage pathways [19^▪^]. For this reason, most of studies on SLN in OC injected the tracer in the infundibulo-pelvic and utero-ovarian ligaments (Table 1). We must report that there is a different methodological approach across published studies. In fact, few studies reported the tracer injection when the ovarian mass was still in situ, before its removal [9^▪▪^,15^▪^,17^▪^]. This potentially allowed the injection directly in the ovarian cortex (or in the capsule of the mass) or in the (sub-peritoneal layer of the) ovarian ligaments but with the risk of performing tracer injection in patients with benign or borderline tumors (not requiring lymph node assessment). The per patient detection rate of SLN in the cases of injection with mass in situ was 91.7% (33/36) [15^▪^,17^▪^]. On the other hand, other studies reported the injection in the stumps of the ligaments, only after adnexectomy with frozen section confirmation of epithelial malignancy or in case of re-staging procedures [13^▪▪^,^14^^▪▪^,16^▪^]. Obviously, utero-ovarian ligament could not be injected if uterus has been previously removed. The SLN detection rate of studies reporting the injection after adnexectomy was 62.8% (132/210) per patient.

The site of injection did not significantly affect detection rate in the meta-analysis from Agusti *et al.*[18^▪^], with a reported detection rate of infundibulo-pelvic injection being 88.5% compared to 92.9% of the utero-ovarian ligament.

An alternative site of injection to utero-ovarian ligament has been proposed by Uccella *et al.* who found that application of tracer into uterine cervix stroma led to the detection of the same lymph node if tracer was applied to utero-ovarian ligament in 18 patients [20^▪^].

The timing between tracer injection and the detection of SLN has been reported between 10 and 15 min in majority of studies, except for those using ICG alone, reporting a shorter waiting time due to transient flow of this tracer [13^▪▪^,^14^^▪▪^,15^▪^–17^▪^].

## DETECTION RATE PER SITE OF INJECTION

SLN detection rate in patients with confirmed ovarian malignancy varied across different studies and ranged between 9.1% and 91.3% for the injection in the utero-ovarian ligament and migration to pelvic lymph nodes and between 27.3% and 100% for the injection in the infundibulo-pelvic ligament and migration to para-aortic lymph nodes [13^▪▪^,^14^^▪▪^,15^▪^–17^▪^] (Table 1). Few studies analyzed the risk factors potentially associated with SLN detection in OC, however they were not able to find any variable associated with mapping failure [13^▪▪^,15^▪^,18^▪^].

## SENTINEL LYMPH NODE RELATED COMPLICATIONS

Intra- and postoperative complications of SLN in OC were reported by few studies [13^▪▪^,15^▪^]. None of them described vascular injuries specifically due to SLN biopsy but they associated these complications with the following systematic lymphadenectomy. One study reported one intra-operative death, but this was not related to tracer injection or the SLN biopsy itself [13^▪▪^]. Lastly, no study reported postoperative complications directly related to SLN biopsy (patients underwent also full lymphadenectomy so postoperative complications could not be attributed to SLN only).

## SENSITIVITY AND ACCURACY

As the sensitivity and accuracy of SLN procedure are still under investigation, the use of SLN biopsy in OC was followed by systematic lymphadenectomy in the published studies [13^▪▪^,^14^^▪▪^,15^▪^–17^▪^]. The sensitivity of SLN in detecting lymphatic metastasis ranged between 73.3% and 100%, while the accuracy was reported to be 96–100% (Table 1). Interestingly, Uccella *et al.* performed a study in which they applied ultrastaging protocol to SLNs as well as to the non-SLNs removed as part of systematic lymphadenectomy [21]. The authors found that out of four SLNs submitted to ultrastaging, one was positive for isolated tumor cells (ITCs) with all other 27 lymph nodes negative at ultrastaging. Author concluded that their report supported SLN concept in OC consistent with the assumption that SLN is the lymph node at highest risk of harboring metastasis.

## ULTRASTAGING METHOD

SLN is analyzed by ultrastaging methodology which consists of performing serial multilevel sections with the use of immunohistochemistry (IHC) to detect the presence of low-volume metastases once the macro-metastases have been excluded by standard hematoxylin and eosin (H&E) staining on bisection. The ultrastaging protocols in OC have been reported to be slightly different in the published studies [13^▪▪^,^14^^▪▪^,15^▪^–17^▪^]. In a study by Lago *et al.*, authors reported in detail the ultrastaging protocol that was used to assess SLN consisting in two sections at each of the 200 μm levels: one that was stained with H&E and one for IHC with cytokeratin AE1/3. Before proceeding with the IHC, the H&E sections were evaluated in order to exclude the presence of macro-metastases. In this setting, the use of SLN represents an essential tool to detect the presence of low-volume metastases (micro-metastases and isolated tumor cells). Interestingly, two studies reported the presence of low-volume metastases in the SLNs to be the only site of lymph node involvement, in a total of 8/20 (40%) of SLN positive patients: these metastases would have been missed without ultrastaging of SLN [13^▪▪^,15^▪^]. Nonetheless, the clinical significance of low-volume lymph node metastasis in OC has not been explored yet.

## COMMENT

The first report on the use of SLN in OC was published in 2014 by Kleppe *et al.* with the use of blue dye and radioactive colloid injected into the proper ovarian ligament and infundibulum-pelvic ligament of the ovary [9^▪▪^]. Since then, few studies have been published using different surgical techniques [18^▪^] both in the setting of primary and re-staging surgery. Type of tracer has changed from radioactive colloid (technetium) with or without blue dye to the use of ICG alone or in combination with radioactive colloid (Table 1) [13^▪▪^,^14^^▪▪^,15^▪^–17^▪^,^22^]. The best SLN detection rates in literature have been reported by those studies using ICG in combination with radioactive colloid [14^▪▪^,15^▪^]. One may hypothesize that this is related to the risk of ICG peritoneal spillage when using ICG alone, which can affect the localization of the SLN. This issue could be partially solved by ICG injection into the sub-peritoneal layer of the infundibulo-pelvic ligament with mass in situ and in the cervix with subsequent transperitoneal identification of SLN [15^▪^,17^▪^,20^▪^,^23^]. By doing this, the ICG spillage should be avoided as retroperitoneal spaces have not been opened yet. However, this approach carries the risk of performing tracer injection in patients with nonmalignant ovarian tumors, with potential vascular injury and allergic reactions. In this context, we have to acknowledge the extremely low incidence of allergic reactions to ICG as reported by Capasso *et al.* in the setting of endometrial cancer [24]. Volume of ICG injected might also represent a risk factor for peritoneal spillage of green dye with higher volume of injection corresponding to higher risk of spillage. Similarly, the radioactive tracer injection before obtaining the frozen section result of the ovarian mass, might also be questioned as a risk for the patients.

As shown in Table 1 and demonstrated by multiple literature reports, the higher risk of SLN metastasis is located in the para-aortic area [13^▪▪^,^14^^▪▪^,^25^]. However, different studies reported the presence of positive SLN in pelvic areas, thus highlighting the importance of uterine tracer (along with infundibulo-pelvic) injection [13^▪▪^,15^▪^]. For the same reason, Table 1 includes SLN detection rate per site of injection rather than per patient. Overall SLN detection rate varies significantly across different studies (from 27.3% [16^▪^] to 100% [14^▪▪^,17^▪^]). However, we need to highlight the fact that majority of reported studies are performed in a single-center setting with the only multicenter study reporting an overall detection rate of 58.6% [13^▪▪^]. The multicenter approach to SLN biopsy studies enables an evaluation of the reproducibility of the surgical technique, which represents a crucial step before introducing it in the daily clinical practice. Of course, only referral centers for gynecologic cancers should be involved.

Accuracy and sensitivity of SLN in representing the lymph node status in apparent early-stage OC were reported to be promising in the published studies with only one study being inferior to 100% [13^▪▪^]. As the therapeutic benefit of systematic lymphadenectomy is still debated [8^▪^], the use of SLN alone to upstage apparent early-stage OC (with adjuvant and maintenance therapy implications) can be considered a visionary approach. Although larger evidence on the accuracy of SLN is needed before SLN alone can be implemented as standard procedure in OC, the high rate of nodal metastasis detected thanks to ultrastaging protocol emphasizes the need for improved prognostic stratification of these patients.

We believe that SLN biopsy will be implemented in the future surgical management of apparent early-stage OC as tool to decrease peri-operative morbidity, increase precision medicine and tailor adjuvant treatment. The best surgical technique to increase SLN detection rate and accuracy is still under investigation.

SLN biopsy can be considered the first step of the future approach to apparent early-stage OC which might include radiomics analysis, liquid biopsy and pre/intra-operative use of cancer-specific tracers, to determine the presence of nodal metastasis [26–28].

## CONCLUSION

SLN biopsy represents a promising tool to assess lymph node status in apparent early-stage OC. The type and volume of injected tracer need to be considered as seem to affect SLN detection rate. Ultrastaging protocol is essential to detect low volume metastasis, the prognostic significance of which has not been determined yet. The detection of low volume metastases by ultrastaging of SLN has unveiled the limitations of traditional staging approaches, raising concerns about their accuracy. Sensitivity and accuracy of SLN biopsy are encouraging, providing tracer injection in both uterine and ovarian ligaments. Integration of SLN biopsy with novel approaches such as radiomics, liquid biopsy and cancer-specific tracers to detect lymph node metastasis represents the future challenge for gynecologic oncologists.

## Acknowledgements

*We would like to thank surgical team and scrub nurses at Policlinico Agostino Gemelli IRCCS, Rome, Italy.*

### Financial support and sponsorship

*This study received no funding or sponsorship.*

### Conflicts of interest

*There are no conflicts of interest.*

## REFERENCES AND RECOMMENDED READING

Papers of particular interest, published within the annual period of review, have been highlighted as:

▪ of special interest
▪▪ of outstanding interest
